# Sex-specific presentation, epidemiology, and control of schistosomiasis in women and adolescent girls

**DOI:** 10.1016/S2214-109X(26)00022-7

**Published:** 2026-05-13

**Authors:** Jane K Maganga, Amina Yussuph, Pamela S Mbabazi, Louis-Albert Tchuem Tchuenté, Amy S Sturt, W Evan Secor, Jennifer A Downs

**Affiliations:** a Mwanza Intervention Trials Unit, National Institute for Medical Research, Mwanza, Tanzania; b Department of Obstetrics and Gynaecology, Bugando Medical Centre, Mwanza, Tanzania; c Department of Control of Neglected Tropical Diseases, World Health Organization, Geneva, Switzerland; d Centre for Schistosomiasis and Parasitology, Faculty of Sciences, University of Yaoundé I, Yaoundé, Cameroon; e Infectious Diseases Section, Veterans Affairs Palo Alto Health Care System, Palo Alto, California, USA; f Division of Infectious Diseases and Geographic Medicine, Department of Medicine, Stanford University, Palo Alto, CA, USA; g Division of Parasitic Diseases and Malaria, Centers for Disease Control and Prevention, Atlanta, GA, USA; h Center for Global Health, Weill Cornell Medicine, New York, NY, USA; i Department of Medicine, Weill Bugando School of Medicine, Mwanza, Tanzania

## Abstract

Schistosomiasis presents a major public health problem for women and adolescent girls in endemic areas, particularly tropical and subtropical regions. Many sex-specific effects of schistosomiasis are due to *Schistosoma haematobium*, which primarily affects the genitourinary tract. Deposition of schistosome eggs in genital tissue causes female genital schistosomiasis (FGS), a chronic gynaecological condition that affects over 40 million women and girls. Women and girls living with FGS face intersecting challenges that compound its adverse effects. Disabling genital symptoms include pain, abnormal discharge, menstrual irregularities, and subfertility. These symptoms affect daily activities, impose financial burdens, and harm intimate relationships. Women and girls face stigma and isolation due to the similarity of FGS symptoms to sexually transmitted infections. Health systems in endemic areas are poorly equipped to consider, diagnose, and treat FGS, and women and girls are often excluded from schistosomiasis control strategies. Multifaceted approaches targeting schistosomiasis in women and girls are urgently needed.

## Introduction

Schistosomiasis is a neglected tropical disease that affects humans throughout their lives. The complex parasitic worm lives for years in blood vessels and lays eggs that have been found in nearly every organ of the body, but that are primarily localised in the urogenital and gastrointestinal tracts. Consideration of the effects of schistosome infections based on host sex is essential, due to sex-specific interactions of the host immune system with parasite antigens^1^ as well as organ-specific damage. Genital tract involvement in women and girls, known as female genital schistosomiasis (FGS), has profound implications for female reproductive health and wellbeing.

This Series paper consolidates the latest scientific research on the effects of schistosomiasis on women and girls, with special attention to the unique sex-specific aspects of the infection. We provide an overview of the parasite, its sex-specific manifestations in women and girls, and schistosomiasis epidemiology and control with particular focus on women and girls. We also consider the lived experiences of women and girls with schistosomiasis. Three accompanying Series papers delve into current FGS diagnostics and treatment, management of schistosomiasis during pregnancy and breastfeeding, and interactions of FGS with co-infections in the genital tract. Some studies have linked *Schistosoma haematobium* infection with HIV-1 and human papillomavirus infections and a recent review highlighted the interactions between HIV-1 and schistosomiasis based on host sex.^1^ This review concluded that women with schistosome infections probably have higher rates of HIV-1 acquisition and death, and that greater HIV-1 transmission has been shown in both women and men with schistosome co-infection. The overarching goal of this Series is to provide a comprehensive resource that will guide research, public health decisions, and clinical care for millions of women and girls affected by *Schistosoma* parasites.

## Epidemiology and prevalence

**Figure 1:**
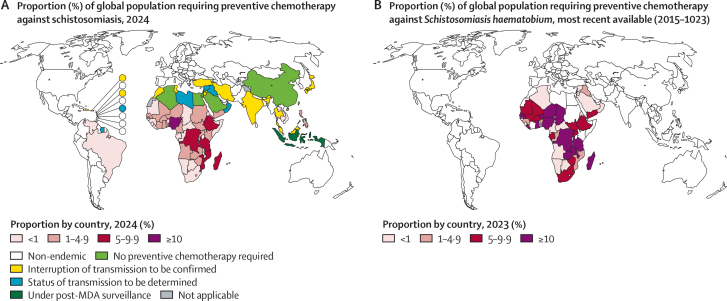
World maps showing the proportion of the global population requiring preventive chemotherapy against schistosomiasis (A) The global distribution of infections caused by all *Schistosoma* species requiring preventive chemotherapy according to country-specific prevalence estimates. Data from WHO.^3^ (B) The global distribution of infections caused by *Schistosoma haematobium* requiring preventive chemotherapy according to country-specific prevalence estimates. Data sources: Global Health Observatory^4^ and Stanford University. Figure created with BioRender.com.

Schistosomiasis transmission has occurred in 78 countries worldwide, primarily in the tropical and subtropical zones. More than 250 million people required preventive chemotherapy in 2024, and schistosomiasis is estimated as responsible for 2·5 million disability-adjusted life years, which could be an underestimate that only accounts for the most severe forms of the disease and does not include genital manifestations.^2^ *S haematobium—*the causative agent for most cases of FGS—is prevalent throughout Africa and in some countries in the eastern Mediterranean (figure 1). *Schistosoma mansoni* is endemic throughout much of sub-Saharan Africa and northeast Brazil, and small pockets are found in Venezuela and Suriname. In the past, *S mansoni* was endemic on several Caribbean islands but, because of economic development, prevalence has decreased sharply in recent years. Efforts to verify if transmission has been interrupted are underway.^5^ Similarly, *Schistosoma japonicum* is close to elimination in several previously endemic areas of China and central Sulawesi, Indonesia. However, it remains quite prevalent in parts of the Philippines.^6^ *Schistosoma mekongi* is present in small areas of northern Cambodia and Laos. *Schistosoma intercalatum* and *Schistosoma guineensis* are present in parts of west and central Africa. In recent years, hybrids of *S haematobium* and veterinary species of schistosomes have been identified in west Africa and in Corsica, France, but the extent and impact of these infections remain unclear.^7^, ^8^

**Key messages**

• The symptoms and signs of female genital schistosomiasis (FGS) are commonly misdiagnosed as sexually transmitted infections
• Comprehensive curricula are needed to equip health workers in endemic and non-endemic areas to diagnose, treat, and prevent FGS
• FGS diagnosis and treatment should be incorporated into sexual and reproductive health care
• Community awareness regarding schistosomiasis and FGS is needed to combat stigma
• Availability of equipment and supplies for schistosomiasis and FGS diagnosis and drugs for treatment must be improved at health facilities in endemic areas
• Governments must increase commitment to providing access to clean water in rural endemic areas
• There is a need for community-based mass drug administration with strategies that will target populations at risk, including women and adolescent girls

The prevalence and intensity of schistosomiasis tend to be highest in primary school-aged children, largely due to their high contact with water containing the larval stage of the parasite (cercariae). However, infections have also been observed in infants and toddlers who are bathed in cercaria-containing water.^9^ In addition, some degree of immunity to reinfection develops in later adolescence and might be partially responsible for the moderate decreases in prevalence observed in older individuals.^10^, ^11^ Nevertheless, adults can develop high-intensity infections—defined as at least 400 eggs per gram of faeces for intestinal schistosomiasis or at least 50 eggs per 10 mL of urine for urinary schistosomiasis^12^—due to freshwater exposure in the setting of domestic chores or occupational exposures such as fishing, agriculture, or sand harvesting.

FGS can occur upon infection with any schistosome species if eggs accumulate in the upper or lower female genital tract but is predominantly associated with *S haematobium* infections.^13^ As with any morbidity associated with schistosome infection, the frequency of FGS is increased with higher prevalence and parasite burden after repeated exposures to contaminated water. However, FGS can occur even in travellers who have much lower levels of exposure to infection than women living in endemic areas.^14^, ^15^ Genital involvement might develop early in young girls, but is most frequently observed in women who have a higher prevalence of genital lesions, probably due to chronic egg deposition from repeated infections throughout their lifetime.^16^

Information on the distribution of FGS is limited to those areas where research studies have been performed and have the equipment and trained personnel needed to perform the diagnosis. As a result, the global burden of FGS is poorly understood; however, conservative estimates predict that over 40 million women and girls are affected, with the highest prevalence occurring in sub-Saharan Africa.^17^ Community-based studies using diagnosis by cervicovaginal biopsy reported a median FGS prevalence of 54% (IQR 21·5–60·5) in women and girls with *S haematobium* infection.^18^ Because these estimates are derived from women who were willing to participate in genital tract studies, they might be an under-representation. A six-country study in Africa using random household sampling is currently underway and will provide the first large-scale estimate of the prevalence and burden of FGS diagnosed by visual examination, symptoms, and antibody testing.^19^

## Lifecycle and transmission

**Figure 2:**
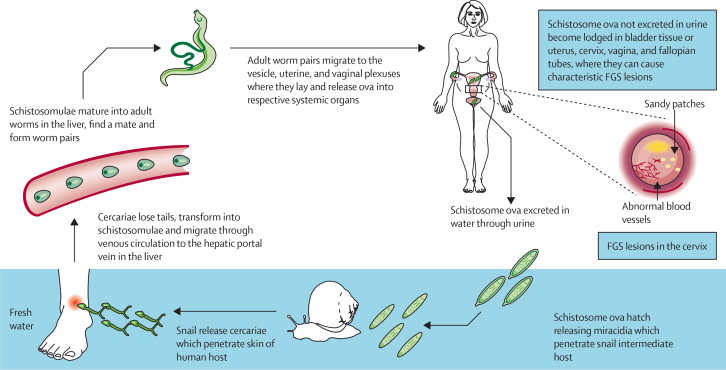
*Schistosoma* life cycle with a focus on *Schistosoma haematobium* in women and girls The snails depicted here transmitting *S haematobium* are from the *Bulinus* genus. *Schistosoma mansoni* is transmitted by *Biomphalaria* snails and *Schistosoma japonicum* is transmitted by *Oncomelania* snails. FGS=female genital schistosomiasis. Figure created with BioRender.com.

Individuals become infected with schistosomes when they contact freshwater containing cercariae released from the species-appropriate intermediate host snail (figure 2). Cercariae penetrate the skin of the definitive host. As the cercaria enters the host, its tail detaches and the head rapidly transforms into a schistosomula.^20^ Over the course of the next 4–6 weeks, the schistosomulae migrate through the host's heart, lungs, and liver as they mature into adult male and female worms.^20^, ^21^ Adults of most schistosome species eventually reside in the mesenteric venules of the portal vein system. However, adult *S haematobium* worms preferentially live in the vesical venous plexus enveloping the bladder, as well as the uterine and vaginal venous plexuses of women.^22^ Male and female worms live in copula (ie, the state of copulation), with female worms producing an estimated 150–3000 eggs per day, depending on the species.^20^ Many of these eggs lodge in host tissues, where they induce the pathology associated with the disease. However, for perpetuation of the lifecycle, eggs must pass from the host into the environment. Host egg excretion is dependent on the anatomical location in which adult worms reside. For *Schistosoma* species that live in the blood vessels near the intestines, eggs pass through the intestinal wall and are excreted in the faeces. However, *S haematobium* eggs pass through the bladder wall and are excreted via urine. If these eggs enter freshwater, they hatch and release a miracidium that seeks out and infects the appropriate intermediate host snail. Inside the snail, the miracidium transforms into a mother sporocyst, giving rise to daughter sporocysts. These eventually give rise to the infectious cercariae.^20^, ^21^ Asexual replication within the snail amplifies the number of parasites and contributes to the challenges of control efforts.

## Development and presentation of FGS

### Pathogenesis

Of the schistosomes, *S haematobium* is the only species that has a predilection for the interconnected uterine, vaginal, and vesicle venous plexuses that drain the bladder and genital organs.^17^, ^22^ Although bladder involvement is found in almost all *S haematobium* infections, autopsy studies indicate that the involvement of genital organs seems more common in infections with the highest parasite burden, affecting an estimated 33–75% of females with *S haematobium* infection.^17^, ^23^ *S mansoni* eggs have also been found in the female genital tract and can cause FGS,^24^ suggesting that worms can move from the anorectal venous plexus into the urogenital venous plexus.^22^ In a full-body autopsy study conducted in Egypt, 24% of detected *S mansoni* eggs were found in the genitourinary organs.^25^ Likewise, case reports have documented the presence of *S japonicum* in the fallopian tube and ovary and hybrid schistosomes (*Schistosoma mattheei*) in the cervix;^26^ however, this evidence is limited and further research is needed.^27^, ^28^

*S haematobium* eggs can be found throughout the female reproductive tract, with the most common sites being the cervix, fallopian tubes, uterus, and vagina.^29^, ^30^ External genitalia, such as the vulva and vagina, are commonly affected in adolescents but are less commonly affected in older women.^31^ Eggs that are migrating through tissue secrete proteins that become targets of the host immune system.^32^ An influx of immune cells, including lymphocytes, eosinophils, neutrophils, and macrophages, surround the eggs to form a granuloma to contain the tissue destruction caused by the proteolytic enzymes released by the eggs. This can subsequently develop into tissue fibrosis.^33^, ^34^ The response to sequestered eggs leads to an altered tissue mucosal immune environment with microscopic and macroscopic mucosal lesions, which together constitute FGS.^33^, ^35^

### Cervicovaginal lesions

**Figure 3:**
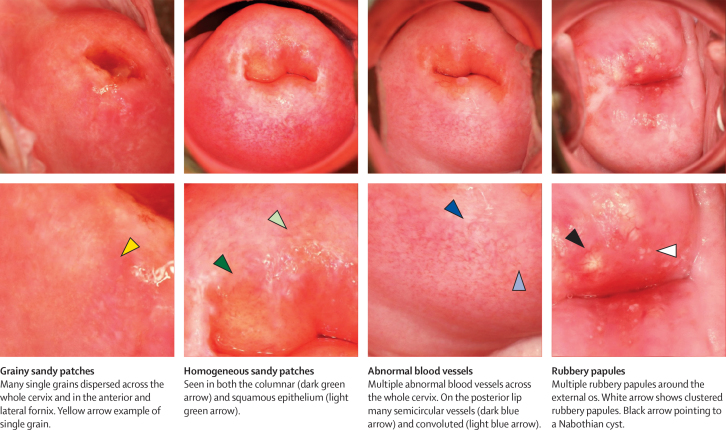
Four characteristic changes on the cervix associated with *Schistosoma haematobium* eggs Four colposcopic images of the cervix (top row) with magnification and arrows denoting areas of abnormality (bottom row). Yellow arrows indicate grainy sandy patches, green arrows indicate homogeneous yellow sandy patches, blue arrows indicate abnormal blood vessels, the white arrow indicates rubbery papules, and the black arrow indicates a Nabothian cyst, which is not associated with female genital schistosomiasis.

*S haematobium* eggs found in genital tissue are associated with cervicovaginal mucosal changes that are visible during gynaecological examination. The association of lesions such as papillomatous tumours, sandy patches, oedema, petechiae, ulcerations, and erosions with biopsy-confirmed FGS has been evaluated in multiple studies.^35^, ^36^, ^37^ The four most common mucosal changes associated with the presence of *S haematobium* eggs are grainy sandy patches (GSP), homogeneous yellow sandy patches (HYSP), rubbery papules, and abnormal blood vessels (figure 3).^38^ In one Zimbabwean study, abnormal blood vessels were the most common finding (231 [44%] of 527 participants).^39^ These vessels are described as pathological, visible within the mucosa (reticular), branched, and of uneven calibre.^39^ GSP were the second most common finding (159 [30%] of 527 participants) and were described as small (0·05 mm × 0·2 mm), oval, non-mobile lesions situated in the cervicovaginal mucosa.^39^ The third most common finding was HYSP (154 [29%] 527 participants). When viewed at 15x magnification, HYSP are yellow patches with no distinct grains.^39^, ^40^ Rubbery papules are small, raised, firm, exophytic, well circumscribed lesions that range from 0·3 mm to 1·2 mm in size.^41^

GSP are thought to be pathognomonic for FGS as they are not associated with sexually transmitted infections (STIs) or cervical precancer.^39^ Of the macroscopic cervicovaginal changes associated with FGS, there is strong evidence from two studies performed in endemic areas (Malawi and Tanzania) that sandy patches are associated with biopsy-proven FGS.^35^, ^36^ However, not every woman who has eggs present in a genital sample has sandy patches, with prevalence of this sign ranging between 27% and 63% in women who test positive for genital eggs. Notably, in the two studies showing an association between biopsy-confirmed FGS and sandy patches, the study authors did not distinguish between GSP and HYSP.^35^, ^36^ A third study, which took place in Zimbabwe, differentiated between GSP and HYSP. This study reported that GSP were associated with *S haematobium* eggs on wet mount and Pap smear, but not with finding eggs on histopathology.^39^ Similarly, HYSP were associated with *S haematobium* eggs on wet mount and Pap smears, but not with eggs in cervical biopsy tissue. Importantly, power in this Zimbabwean study was low because biopsies were performed in only 36 (7%) of 527 women for whom there was concern for malignancy.^39^ Because genital biopsy specimens are intentionally kept small (2·5–5 mm) to minimise patient impact, it is possible that genital eggs were not detected. However, given the lack of association, additional data are needed to confirm if GSP can be considered pathognomonic for FGS. Furthermore, differentiating between GSP and HYSP might be important as HYSP were also associated with high-risk human papillomavirus, herpes simplex virus-type 2, and *Chlamydia trachomatis* and thus are not specific to FGS*.*^39^

Rubbery papules were initially described in Madagascar but have also been documented in Nigeria and Malawi. On biopsy, rubbery papules have been associated with viable parasite eggs and tissue eosinophilia.^41^, ^42^ Notably, in one study, rubbery papules decreased in prevalence with age and with increased intensity of urinary schistosomiasis and thus might be associated with more recent infection.^41^ Rubbery papules are also not specific to FGS and can appear similar to Nabothian cysts.^40^ Although abnormal blood vessels are included in the visual FGS diagnostic criteria, they have only been associated with biopsy-confirmed eggs in one small study in which biopsy specimens of 20 Malawian women with FGS were compared with non-endemic controls.^43^ This study showed that cervical tissue surrounding parasite eggs had a higher density of established blood vessels compared with non-endemic controls (p=0·017), although no differences were seen in comparison to endemic controls without eggs seen in biopsy.^43^ However, abnormal blood vessels have also been associated with cervical precancer.^39^ Importantly, FGS can also occur in macroscopically normal tissue. In 34 (25%) of 134 Tanzanian women with biopsy-proven parasite egg deposition in the cervix, vulva, and vagina, the affected tissues appeared macroscopically normal.^36^

### Symptoms and presentation

Common symptoms ascribed to *S haematobium* infection in both sexes include dysuria, visible haematuria, and lower abdominal pain.^12^ Urogenital symptoms are commonly reported among women in schistosomiasis-endemic areas and include abdominopelvic pain, menorrhagia, genital pruritis, dysmenorrhoea, dyspareunia, and vaginal discharge.^44^ Urogenital symptoms described among women with biopsy-proven FGS include lower abdominal pain,^45^, ^46^ vaginal itching,^46^ dysuria,^36^, ^45^ vaginal discharge,^46^, ^47^ dysmenorrhoea,^47^ and pelvic pain.^47^ The prevalence of these symptoms varied by study site. Most women in Malawi with biopsy-proven FGS reported lower abdominal pain (24 [73%] of 33 women), dysuria (22 [67%] of 33 women), dyspareunia (16 [52%] of 31 women), and menorrhagia (13 [50%] of 26 women).^45^ However, in a Ghanian study, women with biopsy-confirmed FGS complained of vaginal itch (34 [81%] of 42 women), lower abdominal pain (28 [67%] of 42 women), and increased vaginal discharge (24 [58%] of 42 women).^46^ Additionally, in the Ghanian study, vaginal itching (p=0·04) and lower abdominal pain (p=0·04) were associated with biopsy-proven FGS.^46^ Importantly, these associations are not consistent across these small studies and generalisability is unknown.^36^, ^45^ Thus, clinical symptoms are not FGS-specific and cannot be used to differentiate FGS from other clinical entities, including STIs.^47^ In a patient who can be confirmed to have past or present schistosomiasis by microscopy, schistosome antigens, or schistosome antibodies, the positive predictive value of these symptoms for FGS might be higher, but additional research is needed.^48^, ^49^

## Social impacts of health and FGS

### Lived experience

FGS can profoundly impact the health and wellbeing of women and adolescent girls, particularly when they have disabling genital symptoms. Vivid descriptions of feeling “like my vagina has become larger”,^50^ “I just itch myself until it swells and when I move it is so painful”,^51^ and describing sexual intercourse as painful as “a wound that has been touched”^50^ in women who had cervical lesions consistent with FGS provide insight into how FGS can interfere with daily activities. Poor health impairs work productivity and perpetuates the cyclical relationship between poverty and schistosomiasis.^52^ Seeking care is associated with the financial burdens of medical costs and lost work time.^50^ Many women reported spending considerable funds on treatment of presumed STIs. When their symptoms did not resolve with STI treatment, they felt resignation and hopelessness.^50^, ^51^ Similar resignation was expressed by adolescent girls, who attributed menstrual pain and genital symptoms to washing their menstrual cloths in lake water but had no alternative in their community.^53^

FGS might affect a woman's relationship with her partner. Women with severe pain or bleeding during sexual intercourse described being abandoned by angry partners who suspected STIs.^50^, ^53^ Others’ partners remained with them but sought additional sexual partners outside the marriage, which particularly occurred when women with FGS had infertility or recurrent miscarriages.^50^ In Malawi, partners of women with biopsy-proven FGS more frequently had children with other women (p=0·05).^45^ Some women described shame and disappointment, particularly when they were unable to bear children.^53^ The situation might be compounded by women feeling disempowered to explain their feelings to their partners, leading to further estrangement.^50^

### Stigma

Subfertility associated with *S haematobium* has been reported in population and epidemiological mapping studies.^51^, ^54^, ^55^ In many FGS-endemic areas, fertility is prized and a woman's social value is inextricably connected with motherhood.^52^, ^53^ Inability to fulfil this role due to FGS-induced infertility or subfertility has been reported to lower a woman's value to her husband and her social standing in her community.^52^, ^53^ For many women in sub-Saharan Africa, infertility is viewed as more stigmatising than an HIV diagnosis.^56^

Women might additionally lose community respect if FGS-associated disability diminishes their productivity in work and household chores. Furthermore, because symptoms are similar to those caused by STIs, a woman's genital complaints are often assumed to be caused by promiscuous behaviour.^50^, ^53^, ^57^ Women with FGS symptoms describe feeling excluded and unsupported by both relatives and neighbours,^50^, ^53^ leading to additional isolation and distress, particularly for adolescent girls who fear judgment if they report having gynaecological symptoms.^58^ Approximately a third of women in DR Congo stated that they would not share a diagnosis of FGS with their partners and two-thirds would not share their diagnosis with loved ones.^59^

The similarity of symptoms between FGS and STIs can deter women who fear stigma from seeking care. Reports of health-care providers who scolded women—particularly adolescent girls—for being promiscuous when they described genital symptoms are not uncommon.^60^, ^61^ Other affected women feared that even going to the clinic would result in stigma in their small communities.^57^ Some ultimately decided that avoiding stigma was better than daring to seek medical care for FGS symptoms.

### Knowledge gaps

In endemic communities of both west and east Africa, most people are aware of schistosomiasis as a parasitic infection, particularly as it affects children.^57^, ^59^, ^60^ Nonetheless, knowledge gaps persist, and in many communities, few individuals are aware that *S haematobium* infection is associated with genital symptoms.^50^, ^57^, ^59^, ^60^ For instance, only 10·9% of respondents in a Ghanian study^62^ and 45% of respondents in a DR Congo study^59^ were aware of the potential genital involvement and reproductive health outcomes during schistosome infection. Furthermore, many believed FGS is acquired by drinking unclean water or sexual contact.^57^, ^59^, ^60^ Because haematuria is easily visible in boys, the widespread notion that schistosomiasis is a boys’ disease further complicates the community understanding of FGS.^60^

Health-care workers, even those in FGS-endemic areas, also have limited knowledge of genital manifestations. Most drew conclusions based on their knowledge of urinary schistosomiasis and believed that women and girls who are sexually active are at risk of acquiring FGS.^59^, ^60^, ^63^ Perceptions that schistosomiasis predominantly affects children and boys leads many clinicians to omit FGS from the differential diagnosis when a woman presents with gynaecological complaints. Lack of awareness about FGS has also been noted in high-income countries. When 581 physicians across Europe working in fields such as gynaecology, infectious diseases, and family and travel medicine were surveyed in 2023–24, under half were aware of FGS.^64^ Even among those health-care workers with FGS awareness, a lack of proficiency in diagnosing and managing FGS remains a challenge.^65^

In response to these gaps, efforts are ongoing to develop a comprehensive training guide and curriculum that informs health-care workers at all levels in sub-Saharan Africa on how to diagnose, treat, and prevent FGS.^66^ In Ghana, an online interactive training course increased health-care workers’ knowledge and confidence to diagnose and treat FGS.^65^ Similar training courses in Nigeria and Malawi are also underway. Nonetheless, despite these gains, health systems shortcomings remain an issue. Facilities frequently do not have the tools needed for basic schistosomiasis diagnosis and treatment, including urinalysis test strips, microscopes, and praziquantel, which, for example, were reported missing or were inconsistently available in 39–57% of facilities in Nigeria and DR Congo.^59^, ^65^, ^67^ In the absence of mass drug administration (MDA) campaigns, communities in endemic areas have described difficulties in obtaining praziquantel due to inaccessibility, expense, and distance from health facilities. For instance, health-care workers in two endemic districts in Ghana reported situations in which the high cost or unavailability of praziquantel forced affected women to wait for MDA rather than obtaining the medication when it was needed.^65^

## Schistosomiasis control and prevention

To combat the effects of schistosomiasis in the communities where the disease is endemic, WHO recommends access to clean water and sanitation, behavioural change interventions, and preventive chemotherapy by regular treatment through MDA with praziquantel for groups at risk of schistosomiasis as core public health interventions.^2^ The WHO 2021–30 roadmap for neglected tropical diseases aims to eliminate schistosomiasis as a public health problem by reducing the prevalence of high-intensity infections below 1% in all endemic countries.^12^ Groups at highest risk of infection and subsequent morbidity are the primary targets for public health control interventions, with a particular focus on pre-school aged children and adult populations at higher risk of increased parasite exposure, including women of reproductive age and occupational groups at risk.

Pharmaceutical companies play a major role in fighting neglected tropical diseases through donations of essential medicines to WHO.^68^ Since 2007, WHO has received yearly donations of more than 200 million praziquantel tablets from Merck, with the main objective of scaling up interventions to treat as many as 100 million school-aged children per year in sub-Saharan Africa.^69^ These treatments occur largely through school-based distributions. These donation efforts are complemented with comparatively smaller procurements by donors and ministries of health to address the treatment needs of adults. As of 2024, preventive chemotherapy was required for schistosomiasis in 50 countries to treat 134·8 million school-aged children and 118·9 million adults. Although 42% of the countries that reported to WHO are providing preventive chemotherapy to some adults, treatment coverage globally for schistosomiasis in adults remains very low, with only 14·6% of adults who required chemotherapy receiving it.^70^ As of 2024, of 100 countries and territories requiring preventive chemotherapy for at least one neglected tropical disease, only 25 reported some sex-disaggregated data, of which only eight reported on treatments for schistosomiasis. As a result, how many women and girls are being reached effectively with these interventions is largely unknown.^71^

### Sex and age-based gaps in schistosomiasis control

Despite important progress towards schistosomiasis control over the past 20 years, several age groups and subpopulations have been eclipsed by current treatment and monitoring strategies that mainly focus on school-aged children. Schools provide a convenient platform to reach children easily, making this an efficient way to provide MDA.^72^ However, depending on the location and enrolment rate, not all children are in schools or have access to school premises.^73^, ^74^ Furthermore, as girls progress through adolescence, they are less likely to be enrolled and to remain enrolled in schools.^74^ Because school-based MDA is often only provided in primary schools, girls in secondary school might not receive treatment. Girls who miss the benefits of MDA have an increased risk of developing FGS.^75^

Paediatric formulations of praziquantel that are tailored for preschool-aged children have recently been developed, and the European Medicines Agency provided a positive scientific opinion for its use in 2023.^76^ However, with the exception of one project, paediatric formulations are not available for MDA, leaving millions of preschool-aged children at risk.^77^ The new schistosomiasis treatment guidelines recommend treatment for all individuals older than 2 years in endemic areas,^12^ but drug donation programmes are focused on school-aged children. Thus, although schistosomiasis control strategies cover most age groups, the practical application of MDA does not reach other populations at risk, particularly adults.

One consequence of the emphasis on school-aged children in current implementation strategies is that schistosomiasis has received little consideration in gynaecological treatment algorithms, further compounding barriers to treatment access for women affected by FGS.^78^ Widespread lack of awareness among health-care workers about FGS continues to pose a substantial challenge, and few health-care workers in endemic regions have specialised FGS training.^63^, ^65^ This gap is exacerbated by the limited availability of clinical guidelines, diagnostic instruments such as colposcopes, and practical experience with FGS cases in training programmes. The heavy reliance on syndromic management for STIs in low-resource settings leaves health-care providers ill-equipped for recognising and addressing FGS. Additionally, even though praziquantel is included in the essential list of medicines within countries, it is often not in stock within the national health system supply chains. These barriers hinder accurate FGS diagnosis and treatment, perpetuating inadequate management in clinical settings.

To prevent health inequities, schistosomiasis control strategies must urgently be revamped to reach all age groups and to increase access to clinical treatments. Persistent gaps in intervention delivery in endemic areas results in schistosomiasis continuing to pose a serious threat to people of all ages. Ensuring access to praziquantel to all populations by providing MDA through community-based interventions and in clinical settings is essential, especially through platforms that provide reproductive health services for women and girls.^79^ MDA of praziquantel should occur alongside formal inclusion of all age groups in large-scale monitoring and evaluation activities. Together with improving awareness about FGS in neglected tropical disease programmes and among reproductive health-care workers, primary data collection in routine health information systems and periodic disease surveillance systems should be revised to capture information about genital schistosomiasis. Bridging these gaps requires amendments in strategic policy documents to promote integrated approaches for intervention delivery, professional awareness-building initiatives for all levels of health-care providers, and exposure reduction in endemic areas.

### Diagnostic challenges in control programmes for schistosomiasis and FGS

Decisions to initiate or stop MDA are based on schistosomiasis prevalence at the district or subdistrict level. As has been the case for decades, prevalence is determined by detection of parasite eggs in the urine for *S haematobium* and in the stool for all other *Schistosoma* species. Current WHO guidelines recommend annual MDA when egg prevalence in school-aged children is 10% or greater.^12^ However, as prevalence levels decrease in populations and the intensity of infection decreases in individuals, stool and urine microscopy become less sensitive. Recognising the limitations of traditional diagnostic methods for schistosomiasis and other neglected tropical diseases, one of the recommendations of the WHO 2021–30 neglected tropical diseases roadmap was to improve diagnostic tests for these diseases.^2^ To that end, the Diagnostic Technical Advisory Group and disease-specific subgroups were formed.^80^ Part of the mandate for disease-specific subgroups was to generate target product profiles for specific use cases that will help guide diagnostic test developers.

Unlike general schistosomiasis control programmes, diagnosis and control of FGS is challenging and might need to be pursued at an individual level. Ongoing studies are evaluating the association between community *S haematobium* prevalence and the frequency of FGS, with the goal of identifying infection prevalence levels at which FGS no longer occurs. For now, due to resource limitations, the diagnosis of FGS in many endemic settings is dependent on cervical visualisation, ideally with a colposcope, although this instrument is often not available. Cervical visualisation in combination with detection of schistosome eggs, antibodies, or antigen is another possible strategy. Cervicovaginal biopsy is often used when there is concern for malignancy. Given the invasive nature of biopsies, informed consent is required and the risks and benefits of the iatrogenic breach to the cervicovaginal mucosa (including bleeding, pain, and the theoretical risk of HIV and STI acquisition)^81^ should be discussed with the patient. Diagnostic tests for schistosomiasis are addressed in another paper in this Series;^18^ however, until a field-friendly, inexpensive, FGS diagnostic or diagnostic algorithm with optimised operating characteristics is identified, confirmation of FGS in many settings will remain challenging.

Attempts to develop target product profiles for FGS have considered two approaches. The first is to diagnose a woman with *S haematobium* infection and then examine her for FGS if she has gynaecological complaints. The limitation with this approach is that because there is currently no FGS treatment other than treating the underlying schistosome infection with praziquantel, diagnosing the infection first and examining for FGS second will, at present, not change any public health action from that of a general *S haematobium* control programme. The other consideration is to identify women with gynaecological complaints who live in *S haematobium* endemic areas and test them for evidence of schistosome infection. This approach would require introducing schistosome infection into a reproductive health diagnostic algorithm. Syndromic diagnosis and treatment are often used for STIs in low-resource settings,^82^ which not only are ineffective for FGS but also could contribute to increased stigma and antibiotic resistance, and could even result in the unnecessary surgery of women with FGS misdiagnosed with cervical cancer.^75^ Including schistosomiasis in the differential diagnosis for urogenital symptoms might reduce misdiagnosis.

Effective *S haematobium* control should result in lower FGS incidence, and it is hoped that, as girls who benefitted from MDA as school-aged children mature into women, some degree of the burden of FGS will be resolved. One study suggested that adults with *S haematobium* who received treatment during childhood had less prevalent bladder morbidity compared with individuals with no previous treatment.^83^ Similarly, women who received treatment at younger ages (<20 years) were less likely to develop signs of FGS than woman treated later.^84^ Because praziquantel donations focus on MDA for school-aged children, and FGS is often diagnosed in women and adolescent girls, making praziquantel available to individuals at risk of FGS and developing control programmes designed for these populations could help to reduce FGS burden.^75^ Importantly, some, if not most, gynaecological lesions do not resolve even after intensive praziquantel treatment, although gynaecological symptoms improve for many women.^49^, ^85^ Further efforts to optimise existing treatment strategies and identify novel therapies to reverse damage caused by FGS are urgently needed.

## Conclusion

**Figure 4:**
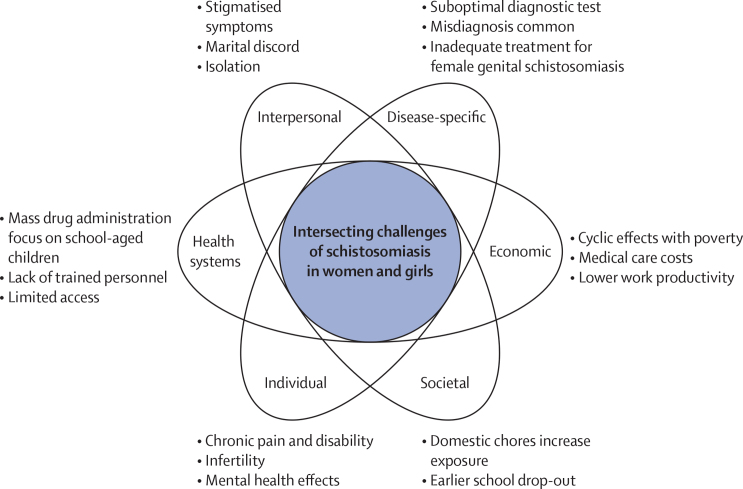
Challenges faced by women and girls living with schistosomiasis Figure created with BioRender.com.

Schistosomiasis in women and adolescent girls represents a locus of intersecting societal and individual challenges. This condition has individual, interpersonal, disease-specific effects together with larger health system, economic, and societal ramifications (figure 4). Priority schistosomiasis-specific challenges in this population include the need for improved diagnostics, the incorporation of FGS into STI and reproductive health algorithms among affected populations, and ensuring that women and adolescent girls benefit from treatment and prevention efforts. Increased awareness of FGS among both health-care providers and affected individuals could improve accurate diagnosis and reduce stigma. High-level challenges include inadequate health systems that are poorly equipped to diagnose and manage FGS, inequitable societal roles for women and girls that increase exposure and decrease access to treatment, and the frequent co-occurrence of schistosomiasis with poverty.

The challenges are stark, but they are not insurmountable. Clinicians, scientists, public health practitioners, community leaders, and patient advocates, among others, have a track record of working together to control severe infectious diseases in the past. With the same cohesion, determination, and perseverance, we can address these challenges in schistosomiasis, improving the health and livelihood for women, girls, and entire communities.

This is the first in a **Series** of four papers about schistosomiasis in women and adolescent girls (papers 2 and 4 appear in *The Lancet Microbe*). All papers in the Series are available at thelancet.com/series/schistosomiasis-in-women-and-adolescent-girls.

### Search strategy and selection criteria

References were identified through PubMed and Ovid MEDLINE searches using the terms “Schistosoma” [Mesh], “schisto*”, “Sex” [Mesh], “Epidemiology” [Mesh], “female genital schistosomiasis”, “stigma”, “age group”, and “praziquantel”. For each section, at least two authors independently generated a list of potential references and compared and discussed references that would best fit for the Series paper. We included articles that were peer-reviewed, written in English, and that reported empirical findings from original research. We included the most relevant articles published between Jan 1, 1950, and April 1, 2025, striving to emphasise the latest research. Additionally, we screened the reference lists of manuscripts found through the literature search and authors’ own article collections. This process yielded more articles than could be included due to space limitations. Therefore, we selected the final reference list based on originality, impact, and overall relevance to the goals of the Series.

### Contributors

## Declaration of interests

## References

[1] Maganga JK, Pham K, Changalucha JM, Downs JA (2025). Sex as a biological variable in HIV-1 and schistosome co-infection. Lancet HIV.

[2] WHO (2020). Ending the neglect to attain the Sustainable Development Goals: a road map for neglected tropical diseases 2021–2030.

[3] WHO Proportion of global population requiring preventive chemotherapy against schistosomiasis, 2024. https://www%.who.int/images/default-source/maps/schistosomiasis_2024.png?sfvrsn=ee49dd06_1.

[4] Global Health Observatory Schistosomiasis. World Health Organization. https://www%.who.int/data/gho/data/themes/topics/schistosomiasis.

[5] Gaspard J, Usey MM, Fredericks-James M (2020). Survey of schistosomiasis in saint lucia: evidence for interruption of transmission. Am J Trop Med Hyg.

[6] Tabilin EJ, Gray DJ, Jiz MA (2025). Schistosomiasis in the Philippines: a comprehensive review of epidemiology and current control. Trop Med Infect Dis.

[7] Boissier J, Grech-Angelini S, Webster BL (2016). Outbreak of urogenital schistosomiasis in Corsica (France): an epidemiological case study. Lancet Infect Dis.

[8] Agniwo P, Savassi BAES, Boissier J, Dolo M, Ibikounlé M, Dabo A (2024). Mapping of schistosome hybrids of the *haematobium* group in west and central Africa. J Helminthol.

[9] Osakunor DNM, Woolhouse MEJ, Mutapi F (2018). Paediatric schistosomiasis: what we know and what we need to know. PLoS Negl Trop Dis.

[10] Mbanefo EC, Huy NT, Wadagni AA, Eneanya CI, Nwaorgu O, Hirayama K (2014). Host determinants of reinfection with schistosomes in humans: a systematic review and meta-analysis. PLoS Negl Trop Dis.

[11] Mutapi F, Burchmore R, Mduluza T, Midzi N, Turner CMR, Maizels RM (2008). Age-related and infection intensity-related shifts in antibody recognition of defined protein antigens in a schistosome-exposed population. J Infect Dis.

[12] WHO WHO guideline on control and elimination of human schistosomiasis. Feb 14, 2022. https://www%.who.int/publications/i/item/9789240041608.

[13] Bustinduy AL, Randriansolo B, Sturt AS (2022). An update on female and male genital schistosomiasis and a call to integrate efforts to escalate diagnosis, treatment and awareness in endemic and non-endemic settings: the time is now. Adv Parasitol.

[14] Helling-Giese G, Demarta-Gatsi C, Richter J (2024). Female genital schistosomiasis (FGS) in returned travellers—a review of reported cases. Eur J Obstet Gynecol Reprod Biol.

[15] Pedeboy D (2023). Female genital schistosomiasis: my personal account and key recommendations to the global health community. Int Health.

[16] Lamberti O, Kayuni S, Kumwenda D (2024). Female genital schistosomiasis burden and risk factors in two endemic areas in Malawi nested in the Morbidity Operational Research for Bilharziasis Implementation Decisions (MORBID) cross-sectional study. PLoS Negl Trop Dis.

[17] Hotez PJ, Engels D, Gyapong M, Ducker C, Malecela MN (2019). Female genital schistosomiasis. N Engl J Med.

[18] Sturt AS, Randrianasolo B, Downs JA (2026). Diagnosis and treatment of female genital schistosomiasis. Lancet Microbe.

[19] Global Schistosomiasis Alliance Research Links | MAP-FGS study insights. March 5, 2025. https://www%.eliminateschisto.org/news-events/events/research-links-map-fgs-study-insights.

[20] LoVerde PT (2024). Schistosomiasis. Adv Exp Med Biol.

[21] Loker E, Hofkin B (2015). Parasitology: a conceptual approach.

[22] Nation CS, Da’dara AA, Marchant JK, Skelly PJ (2020). Schistosome migration in the definitive host. PLoS Negl Trop Dis.

[23] Edington GM, Nwabuebo I, Junaid TA (1975). The pathology of schistosomiasis in Ibadan, Nigeria with special reference to the appendix, brain, pancreas and genital organs. Trans R Soc Trop Med Hyg.

[24] Chaves E, Palitot P (1964). Pelvic schistosomiasis. Am J Obstet Gynecol.

[25] Cheever AW, Kamel IA, Elwi AM, Mosimann JE, Danner R (1977). *Schistosoma mansoni* and *S. haematobium* infections in Egypt. II. Quantitative parasitological findings at necropsy. Am J Trop Med Hyg.

[26] Kayuni SA, Cunningham LJ, Kumwenda D (2024). Challenges in the diagnosis and control of female genital schistosomiasis in sub-Saharan Africa: an exemplar case report associated with mixed and putative hybrid schistosome infection in Nsanje district, southern Malawi. Front Trop Dis.

[27] Carpenter CB, Mozley PD, Lewis NG (1964). *Schistosomiasis Japonica* involvement of the female genital tract. JAMA.

[28] Lee KF, Hsueh S, Tang MH (2000). Schistosomiasis of the ovary with endometriosis and corpus hemorrhagicum: a case report. Chang Gung Med J.

[29] Gelfand M, Ross MD, Blair DM, Weber MC (1971). Distribution and extent of schistosomiasis in female pelvic organs, with special reference to the genital tract, as determined at autopsy. Am J Trop Med Hyg.

[30] Poggensee G, Feldmeier H (2001). Female genital schistosomiasis: facts and hypotheses. Acta Trop.

[31] WHO Report of an informal working group meeting on urogenital schistosomiasis and HIV infection. Feb 12, 2010. https://www%.who.int/publications/i/item/WHO-HTM-NTD-PCT-2010.5.

[32] Ashton PD, Harrop R, Shah B, Wilson RA (2001). The schistosome egg: development and secretions. Parasitology.

[33] Costain AH, MacDonald AS, Smits HH (2018). Schistosome egg migration: mechanisms, pathogenesis and host immune responses. Front Immunol.

[34] Pearce EJ, MacDonald AS (2002). The immunobiology of schistosomiasis. Nat Rev Immunol.

[35] Helling-Giese G, Sjaastad A, Poggensee G (1996). Female genital schistosomiasis (FGS): relationship between gynecological and histopathological findings. Acta Trop.

[36] Poggensee G, Kiwelu I, Weger V (2000). Female genital schistosomiasis of the lower genital tract: prevalence and disease-associated morbidity in northern Tanzania. J Infect Dis.

[37] Randrianasolo BS, Jøker K, Arenholt LTS (2024). An assessment of gynecological manifestations in women with female genital schistosomiasis with reference to *Schistosoma* biomarkers, sexually transmitted infections and bacterial vaginosis. Front Trop Dis.

[38] WHO (2015). Female genital schistosomiasis: a pocket atlas for clinical health care professionals. https://apps.who.int/iris/bitstream/handle/10665/180863/9789241509299_eng.pdf?sequence=1&isAllowed=y.

[39] Kjetland EF, Ndhlovu PD, Mduluza T (2005). Simple clinical manifestations of genital *Schistosoma haematobium* infection in rural Zimbabwean women. Am J Trop Med Hyg.

[40] Norseth HM, Ndhlovu PD, Kleppa E (2014). The colposcopic atlas of schistosomiasis in the lower female genital tract based on studies in Malawi, Zimbabwe, Madagascar and South Africa. PLoS Negl Trop Dis.

[41] Randrianasolo BS, Jourdan PM, Ravoniarimbinina P (2015). Gynecological manifestations, histopathological findings, and schistosoma-specific polymerase chain reaction results among women with *Schistosoma haematobium* infection: a cross-sectional study in Madagascar. J Infect Dis.

[42] Ekpo U, Odeyemi O, Sam-Wobo S (2017). Female genital schistosomiasis (FGS) in Ogun state, Nigeria: a pilot survey on genital symptoms and clinical findings. Parasitol Open.

[43] Jourdan PM, Roald B, Poggensee G, Gundersen SG, Kjetland EF (2011). Increased vascularity in cervicovaginal mucosa with *Schistosoma haematobium* infection. PLoS Negl Trop Dis.

[44] Downs JA, Mguta C, Kaatano GM (2011). Urogenital schistosomiasis in women of reproductive age in Tanzania's Lake Victoria region. Am J Trop Med Hyg.

[45] Kjetland EF, Poggensee G, Helling-Giese G (1996). Female genital schistosomiasis due to *Schistosoma haematobium*. Clinical and parasitological findings in women in rural Malawi. Acta Trop.

[46] Yirenya-Tawiah D, Amoah C, Apea-Kubi KA (2011). A survey of female genital schistosomiasis of the lower reproductive tract in the volta basin of Ghana. Ghana Med J.

[47] Leutscher P, Raharisolo C, Pecarrere JL (1997). *Schistosoma haematobium* induced lesions in the female genital tract in a village in Madagascar. Acta Trop.

[48] Galappaththi-Arachchige HN, Holmen S, Koukounari A (2018). Evaluating diagnostic indicators of urogenital *Schistosoma haematobium* infection in young women: a cross sectional study in rural South Africa. PLoS One.

[49] Arenholt LTS, Randrianasolo BS, Rabozakandraina TOO (2024). Repeated versus single praziquantel dosing regimen in treatment of female genital schistosomiasis: a phase 2 randomised controlled trial showing no difference in efficacy. Front Trop Dis.

[50] Schuster A, Randrianasolo BS, Rabozakandraina OO, Ramarokoto CE, Brønnum D, Feldmeier H (2022). Knowledge, experiences, and practices of women affected by female genital schistosomiasis in rural Madagascar: a qualitative study on disease perception, health impairment and social impact. PLoS Negl Trop Dis.

[51] Masong MC, Wepnje GB, Marlene NT (2021). Female genital schistosomiasis (FGS) in Cameroon: a formative epidemiological and socioeconomic investigation in eleven rural fishing communities. PLoS Glob Public Health.

[52] Vlassoff C, Arogundade K, Patel K, Jacobson J, Gyapong M, Krentel A (2022). Improving the response of health systems to female genital schistosomiasis in endemic countries through a gender-sensitive human rights-based framework. Diseases.

[53] Masong MC, Mengue MT, Marlene NT (2024). Illness experiences and mental health challenges associated with female genital schistosomiasis in Cameroon: a gender analysis. Int Health.

[54] Woodall PA, Kramer MR (2018). Schistosomiasis and infertility in east Africa. Am J Trop Med Hyg.

[55] Miller-Fellows SC, Howard L, Kramer R (2017). Cross-sectional interview study of fertility, pregnancy, and urogenital schistosomiasis in coastal Kenya: documented treatment in childhood is associated with reduced odds of subfertility among adult women. PLoS Negl Trop Dis.

[56] Lawson K, Zablotska-Manos I (2024). Social impacts experienced by women with HIV and infertility in sub-Saharan Africa: a scoping review. Int J STD AIDS.

[57] Mazigo HD, Samson A, Lambert VJ (2021). “We know about schistosomiasis but we know nothing about FGS”: A qualitative assessment of knowledge gaps about female genital schistosomiasis among communities living in *Schistosoma haematobium* endemic districts of Zanzibar and northwestern Tanzania. PLoS Negl Trop Dis.

[58] Mchome Z, Richards E, Nnko S, Dusabe J, Mapella E, Obasi A (2015). A ‘mystery client’ evaluation of adolescent sexual and reproductive health services in health facilities from two regions in Tanzania. PLoS One.

[59] Wambui CW, Madinga J, Ashepet MG, Anyolitho MK, Mitashi P, Huyse T (2024). Knowledge, attitudes and practices toward female genital schistosomiasis among community women and healthcare professionals in Kimpese region, Democratic Republic of Congo. PLoS Negl Trop Dis.

[60] Kukula VA, MacPherson EE, Tsey IH, Stothard JR, Theobald S, Gyapong M (2019). A major hurdle in the elimination of urogenital schistosomiasis revealed: identifying key gaps in knowledge and understanding of female genital schistosomiasis within communities and local health workers. PLoS Negl Trop Dis.

[61] Odhiambo GO, Musuva RM, Atuncha VO (2014). Low levels of awareness despite high prevalence of schistosomiasis among communities in Nyalenda informal settlement, Kisumu city, western Kenya. PLoS Negl Trop Dis.

[62] Yirenya-Tawiah DR, Ackumey MM, Bosompem KM (2016). Knowledge and awareness of genital involvement and reproductive health consequences of urogenital schistosomiasis in endemic communities in Ghana: a cross-sectional study. Reprod Health.

[63] Mazigo HD, Samson A, Lambert VJ (2022). Healthcare workers’ low knowledge of female genital schistosomiasis and proposed interventions to prevent, control, and manage the disease in Zanzibar. Int J Public Health.

[64] Marchese V, Remkes A, Kislaya I (2025). Awareness and knowledge regarding female genital schistosomiasis among European healthcare workers: a cross-sectional online survey. Global Health.

[65] Gyapong M, Dalaba MA, Immurana M (2024). Breaking the silence of female genital schistosomiasis in Ghana's health system: a case of health workers within the FAST project. PLoS Negl Trop Dis.

[66] Jacobson J, Pantelias A, Williamson M (2022). Addressing a silent and neglected scourge in sexual and reproductive health in sub-Saharan Africa by development of training competencies to improve prevention, diagnosis, and treatment of female genital schistosomiasis (FGS) for health workers. Reprod Health.

[67] Aribodor OB, Mogaji HO, Surakat OA (2023). Profiling the knowledge of female medical/para-medical students, and expertise of health care professionals on female genital schistosomiasis in Anambra, south eastern Nigeria. PLoS Negl Trop Dis.

[68] WHO Donated medicines and health products by disease and donor commitment. February, 2025. https://cdn.who.int/media/docs/default-source/ntds/neglected-tropical-diseases-non-disease-specific/ntd-medicine-donation.pdf?sfvrsn=24d10542_23.

[69] WHO New agreement expands access to schistosomiasis treatment for millions. Feb 12, 2013. https://www%.who.int/news/item/12-02-2013-new-agreement-expands-access-to-schistosomiasis-treatment-for-millions.

[70] WHO Schistosomiasis and soil transmitted helminthiases: progress report, 2023. Nov 29, 2024. https://iris.who.int/server/api/core/bitstreams/76aaa8c9-77f4-4a74-b42c-0857f2d936c6/content.

[71] Global Health Observatory (2024). Schistosomiasis. World Health Organization. https://www%.who.int/data/gho/data/themes/topics/schistosomiasis.

[72] Kokaliaris C, Garba A, Matuska M (2022). Effect of preventive chemotherapy with praziquantel on schistosomiasis among school-aged children in sub-Saharan Africa: a spatiotemporal modelling study. Lancet Infect Dis.

[73] Ezezika O, Olorunbiyi O, Gong J, Surakat O, Ogoji J, Nebe O (2025). Implementation of school-based mass drug administration of praziquantel in Nigeria: barriers, facilitators and opportunities for improvement. Glob Health J.

[74] Burnim M, Ivy JA, King CH (2017). Systematic review of community-based, school-based, and combined delivery modes for reaching school-aged children in mass drug administration programs for schistosomiasis. PLoS Negl Trop Dis.

[75] UNAIDS No more neglect: female genital schistosomiasis and HIV: integrating sexual and reproductive health interventions to improve women's lives. World Health Organization. Dec 12, 2019. https://www%.who.int/publications/i/item/UNAIDS-JC2979.

[76] European Medicines Agency Arpraziquantel—opinion on medicine for use outside EU. Dec 14, 2023. https://www%.ema.europa.eu/en/opinion-medicine-use-outside-EU/human/arpraziquantel.

[77] Pediatric Praziquantel Consortium First preschool-aged child receives arpraziquantel for the treatment of schistosomiasis. March 4, 2025. https://www%.pediatricpraziquantelconsortium.org/sites/ppc/files/2025-03/PediatricPraziquantelConsortiumPressRelease_4March2025_ENG.pdf.

[78] WHO (2022). Global health sector strategies on, respectively, HIV, viral hepatitis and sexually transmitted infections for the period 2022–2030.

[79] Engels D, Hotez PJ, Ducker C (2020). Integration of prevention and control measures for female genital schistosomiasis, HIV and cervical cancer. Bull World Health Organ.

[80] Souza AA, Ducker C, Argaw D (2021). Diagnostics and the neglected tropical diseases roadmap: setting the agenda for 2030. Trans R Soc Trop Med Hyg.

[81] Diouf K, Sawaya GF, Shiboski S (2011). Investigating potential associations between cervical procedures and HIV acquisition. ISRN Obstet Gynecol.

[82] WHO Guidelines for the management of symptomatic sexually transmitted infections. July 15, 2021. https://www%.who.int/publications/i/item/9789240024168.

[83] Ouma JH, King CH, Muchiri EM (2005). Late benefits 10-18 years after drug therapy for infection with *Schistosoma haematobium* in Kwale district, coast province, Kenya. Am J Trop Med Hyg.

[84] Kjetland EF, Ndhlovu PD, Kurewa EN (2008). Prevention of gynecologic contact bleeding and genital sandy patches by childhood anti-schistosomal treatment. Am J Trop Med Hyg.

[85] Kabengele C, Mwangelwa S, Kilembe W (2024). Female genital schistosomiasis lesion resolution post-treatment with praziquantel in Zambian adults. Am J Trop Med Hyg.

